# Associations of Critical Relationships With Distress and Burden in Caregivers of Patients With Brain Tumor

**DOI:** 10.1002/pon.70332

**Published:** 2025-11-06

**Authors:** Maija Reblin, Kristen J. Wells, Bradley J. Zebrack, Deanna Witte, Margaret M. Byrne

**Affiliations:** ^1^ Department of Family Medicine University of Vermont Burlington Vermont USA; ^2^ Department of Psychology San Diego State University San Diego California USA; ^3^ School of Social Work University of Michigan Ann Arbor Michigan USA; ^4^ Department of Health Outcomes & Behavior Moffitt Cancer Center Florida Tampa USA

**Keywords:** cancer, caregiver, distress, oncology, social relationship, social support, stress

## Abstract

**Background:**

Little data exists on critical relationships for caregivers of patients with brain tumors, or how their social context impacts caregiver well‐being.

**Aims:**

Our goal was to describe and categorize the critical relationships reported by caregivers of patients with brain tumors, and to assess the association of categories of critical social relationships with caregiver reports of anxiety, depression, and burden.

**Methods:**

We analyzed baseline self‐report data collected from neuro‐oncology caregivers enrolled in a supportive care trial. Data included demographics, presence of critical relationships—a confidante or person contributing stress, anxiety, depression, and burden. Critical relationships categories were operationalized as: positive (confidant, no stressful person), negative (no confidant, stressful person), neutral (no confidant nor stressful person), and ambivalent (confidant and stressful person). Descriptive statistics were calculated. One‐way ANOVAs assessed differences between relationship category groups on caregiver reports of anxiety, depression, and burden.

**Results:**

Data from 119 caregivers were analyzed. Caregivers had a mean age of 57 years (SD = 15), were mostly non‐Hispanic White (92%), female (70%), spouses (52%) of patients with grade 4 tumors (69%). Most caregivers were categorized as having positive (53%) or ambivalent (30%) critical relationships. Significant differences between relationship categories were seen in anxiety, depression, and burden (ps < 0.01).

**Conclusions:**

Although most caregivers of people with brain tumors have confidants, many also identify people who contribute stress. The presence of stressful individuals can negatively impact caregiver well‐being in a way that is not counterbalanced by the co‐presence of a confidant. Future work should focus on helping caregivers manage stressful relationships.

**Study Registration:**

The study was pre‐registered at ClinicalTrials.gov: NCT04268979.

**Analytic Plan Pre‐registration:**

The analysis plan for this manuscript was not formally pre‐registered.

## 1 Background

Family members and friends of patients with brain tumors play a critical unpaid caregiving role, offering emotional support and help with decision‐making and care coordination. Additionally, many forms of malignant brain tumors progress rapidly and have poor prognoses [1]. As the cancer progresses, neuro‐oncology patients tend to have increasing functional dependency as well as cognitive and behavioral changes which require caregiver assistance [2]. Although family caregivers often find meaning in their role, these demands, along with the existential feelings of loss due to changes in their relationship and potential death of the patient, can result in high levels of caregiver burden and distress [2].

Research in neuro‐oncology family caregivers and in oncology caregiving more broadly suggests that, aligned with the Stress Process Model of Caregiving [3], adequate support can protect against these negative caregiver outcomes [4], and in fact better quality caregiver support may be associated with better caregiver and patient health [5, 6]. However, caregiver support can be complex due to the nature of the stressor. In addition to support and collaboration in managing the patient's cancer, caregivers also need support to manage the stress associated with taking on the care role. This can include feelings of obligation, guilt, psychological burden, and role strain. Caregivers may not want to be seen as complaining or competing with the stress of being a cancer patient [36], but this inability or unwillingness to discuss their own struggles or needs can inhibit important processing [7]. As such, the presence of a confidant—someone trusted with whom to share personal information and gain support—may be especially important in this context. Despite this, most research on confidants in the context of cancer family caregivers focuses on the caregiver role as confidant for patients, rather than exploring caregivers' own confidants [8]. Some research suggests that spouse cancer caregivers are 39%–57% less likely to report having a confidant compared to patients, and were 3–4 times less likely to report confiding in their spouse [9]. Research on older adults, who are more likely than younger adults to be caregivers, suggests having at least one confidant is associated with better mental and physical health outcomes [10, 11].

Research on the structural and functional aspects of relationships of older adults with chronic illness suggests that, while having a confidant is strongly predictive of better quality of life, avoiding negative aspects of close relationships was an even stronger predictor [12]. Caregiving may have a negative impact on close relationships and create additional conflict within families [13]. This conflict can include disagreements about the patient's care plan, but can also include secondary stressors around work, household management, or having to negotiate or manage relationships and roles; both primary and secondary stress can negatively impact caregiver outcomes [14, 15]. Research on caregivers of cancer patients enrolled in hospice shows that almost half of caregivers had social networks with high levels of stress in their family [16]. However, despite the powerful role social stress can have in compounding caregiver burden and negatively impacting quality of life, less research has focused on identifying sources of social stress in cancer caregivers [17].

Research on neuro‐oncology caregivers' social networks showed high variability in the number of ties and the amount of support provided [18], but little data exists on the existence of confidants and those contributing social stress, or how these relationships can impact caregiver distress and burden. Prior research from the broader relationships and health literature suggests that supportive and stressful social network ties have different relationship dynamics and positivity and negativity can co‐occur [19]. Additionally, the perception of one of these relationships in a caregivers' social network may change based on the presence of the other. For example, someone contributing social stress may be seen as even more stressful when contrasted with the support of a confidant, or the presence of a confidant may mitigate the impact of that stressful person. These dynamics have not been well explored in neuro‐oncology settings.

Our objective was to describe and categorize the critical relationships reported by caregivers of patients with brain tumors, and to assess the association of categories of critical social relationships with caregiver reports of anxiety, depression, and burden.

## 2 Methods

We conducted a secondary analysis of cross‐sectional self‐report data collected from neuro‐oncology caregivers in the baseline assessment at enrollment of a supportive care trial, as described elsewhere [20]. This study was performed in line with the principles of the Declaration of Helsinki. Approval was granted by the Advarra Institutional Review Board (Pro00029204) prior to study implementation. Informed consent was obtained from all individual participants included in the study.

### 2.1 Participants and Setting

Patient and caregiver participants were recruited as a convenience sample from the neuro‐oncology clinic of a large National Cancer Institute (NCI)‐designated Comprehensive Cancer Center in Florida from February 2020 to June 2024. Patients were screened for potential eligibility and were approached in clinic or by phone; interested patients were asked to nominate a caregiver for participation, who was also approached in clinic or by phone.

Inclusion criteria were: (1) age ≥ 21 years, (2) English‐speaking, (3) able to complete questionnaires (including by proxy). Caregivers self‐identify as being a primary caregiver of a patient with a brain tumor. Patients were within 9 months of diagnosis of a new or recurrent brain tumor (primary or secondary), undergoing active treatment (i.e., had an ongoing clinical relationship, not a second‐opinion consultation), with a prognosis of at least 6 months, and a Karnofsky Performance Status (KPS) score ≥ 50 (determined by oncologist).

### 2.2 Measures

All consented participants completed baseline questionnaires, including demographics, upon enrollment. In this analysis, we focus only on caregiver data.

We identified the presence of confidants and people contributing social stress based on responses to two items from the Duke Social Support and Stress Scale [21]. These items were: “Do you have one particular person whom you trust and to whom you can go with personal difficulties?” and “Is there one particular person who is causing you the most personal stress now?” Responses were either yes or no, and if yes, for each item participants were asked to identify who that person was (naming a role, e.g., spouse, sibling, etc.).

Caregiver burden was assessed using the valid and reliable 12‐item Zarit Burden Interview (ZBI) Short Form [22]. Responses, assessed on a scale of 0–4, are summed to calculate a total score, with scores of 10–20 indicating mild to moderate burden, and scores over 20 indicating high burden. Cronbach's alpha was calculated at 0.904.

Anxiety was measured using the seven‐item valid and reliable Generalized Anxiety Disorder scale (GAD‐7) [23]. Responses, assessed on a scale of 0–3, are summed to create a total score ranging from 0 to 21, with a score of five to nine indicating mild anxiety, 10‐14 moderate anxiety, and 15‐21 severe anxiety. Cronbach's alpha was calculated at 0.920.

Depressive symptoms were measured using the valid and reliable eight‐item Patient Health Questionnaire‐8 (PHQ‐8) [24]. The PHQ‐8 omits a question about suicidality from the PHQ‐9. Responses, assessed on a scale of 0–3, are summed to create a total score. Scores of five to nine indicate mild depression, 10–14 indicate moderate depression, 15–19 indicate moderately severe depression, and 20–24 indicate severe depression. Cronbach's alpha was calculated at 0.884.

### 2.3 Analysis

Using Social Support and Stress Scale items, we created critical relationship categories based on the identification of confidant and stress relationships. Categories included positive (confidant, no stressful person), negative (no confidant, but stressful person), neutral (no confidant, no stressful person), and ambivalent (confidant, and stressful person).

Descriptive statistics were calculated for demographics and critical relationship categories. We also assessed frequencies of the relationships identified as being a confidant or a person contributing stress. Relationships identified as confidant or person contributing stress were also coded to determine if the patient was identified in that role (i.e., spouse was listed as confidant; patient relationship is identified as spouse). One‐way ANOVAs assessed overall differences between critical relationship category groups on demographic data and caregiver reports of burden, anxiety, and depressive symptoms. Tukey HSD post hoc comparisons were conducted to identify differences between specific category groups.

## 3 Results

As presented elsewhere [20], of 502 eligible dyads who were approached, 148 consented to participate (29%), exceeding the median rate (23%) of prior oncology dyadic research [25]. One hundred and nineteen caregivers had complete data at baseline, which was used in the current analysis. Caregivers had a mean age of 57 years (SD = 15), were mostly non‐Hispanic White (92%), female (70%), and spouses (52%) of patients with grade 4 tumors (69%). The median time from patient diagnosis to dyad study consent was 97 days. Additional demographic data are shown in Table 1.

**TABLE 1:** Demographic information.

		Caregivers (*n* = 119)	Patients (*n* = 119)
Variable	Levels	*M* (SD)	*M* (SD)
Age (years)		57.0 (14.4)	58.3 (15.2)

		*N* (%)	*N* (%)
Gender	Male	36 (30%)	67 (56%)
	Female	83 (70%)	44 (37%)
	Missing	0 (0%)	8 (7%)
Race	American Indian or Alaskan native	2 (2%)	0 (0%)
	Asian	0 (0%)	3 (3%)
	Black or African American	4 (3%)	7 (6%)
	Middle Eastern or North African	1 (1%)	0 (0%)
	Native Hawaiian or other Pacific Islander	0 (0%)	1 (1%)
	White or caucasian	111 (93%)	99 (83%)
	Some other race, ethnicity, or origin	4 (3%)	2 (2%)
	Prefer not to answer	1 (1%)	2 (2%)
Ethnicity	Non‐hispanic	111 (93%)	107 (90%)
	Hispanic	7 (6%)	4 (3%)
	Prefer not to answer	1 (1%)	0 (0%)
Education	Less than high school diploma/equivalent	1 (1%)	2 (2%)
	High school diploma or equivalent	16 (13%)	13 (11%)
	Some college	35 (30%)	43 (36%)
	Four‐year college degree	35 (30%)	27 (24%)
	Postgraduate degree	32 (27%)	26 (22%)
	Other	0 (0%)	1 (1%)
	Prefer not to answer	0 (0%)	
Work hours per week	≥ 35 h	40 (34%)	
	< 35 h	9 (8%)	
	Not working for pay	68 (57%)	
	Prefer not to answer	2 (2%)	
Financial situation	Not very good	19 (16%)	
	Comfortable	68 (57%)	
	More than adequate to meet needs	27 (23%)	
	Prefer not to answer	5 (4%)	
Relationship to patient	Spouse/partner	95 (80%)	
	Parent/in‐law	8 (7%)	
	Sibling/in‐law	2 (2%)	
	Child	9 (8%)	
	Friend/neighbor	2 (2%)	
	Other	3 (3%)	
Length of care for patient	1–3 months	31 (26%)	
	4–6 months	33 (28%)	
	7–12 months	19 (16%)	
	1–2 years	14 (12%)	
	3–5 years	13 (11%)	
	More than 5 years	7 (6%)	
	Missing	2 (2%)	
Caregiving hours per week	0	5 (4%)	
	1–5	21 (18%)	
	6–15	23 (19%)	
	16–30	26 (22%)	
	31–50	17 (14%)	
	More than 50	20 (17%)	
	Unable to quantify/Missing	8 (7%)	
Patient diagnosis	Glioblastoma		80 (67%)
	Astrocytoma		9 (8%)
	Oligodendroglioma		4 (3%)
	Glioma NOS		5 (4%)
	Meningioma		6 (5%)
	Medulloblastoma		1 (1%)
	Brain metastasis		14 (12%)
Recurrent brain tumor	No		101 (85%)
	Yes		18 (15%)
Patient tumor WHO grade	1		1 (1%)
	2		6 (5%)
	3		12 (10%)
	4		82 (69%)
	Not in medical record/Not applicable/missing		16 (13%)

			*M* (SD)
Patient cognitive function (Neuro‐QOL: Range 8–40)*			25.7 (9.3)
Patient mobility (Neuro‐QOL: Range 8–40)*			32.8 (7.7)

*Note:* * missing data from 5 patients for cognitive functioning and 6 patients for mobility. Lower scores indicate lower function/mobility.

Confidants were identified by 99 caregivers (83%), and people contributing stress were identified by 42 caregivers (35%). No individual named as a confidant was also identified as a person contributing stress. Of those listing a confidant, most were identified as friends (*n* = 33, 34%), spouse/partners (*n* = 19, 20%), or children (*n* = 14, 14%). Confidant relationships were matched with the patient relationship 11% of the time (*n* = 11), and most often were spouses (*n* = 9). Of those listing a stressful person, most were identified as spouse/partners (*n* = 17, 41%), parents/parents‐in‐law (*n* = 12, 29%), or children (*n* = 5, 12%). Stressful relationships were matched with the patient relationships half the time (*n* = 21), and were also most often spouses (*n* = 15). The most common critical relationship category was positive (*n* = 63, 53%), followed by ambivalent (*n* = 36, 30%), neutral (*n* = 14, 12%), and negative (*n* = 6, 5%). No differences were seen in category membership by caregiver age (*F* = 0.262, df = 3,112, *p* = 0.85), gender (*Χ* ^2^ = 2.869, df = 3, *p* = 0.412), relationship to patient (*Χ* ^2^ = 11.527, df = 15, *p* = 0.714), or amount of time spent providing care (*F* = 1.03, df = 3,91, *p* = 0.365).

**FIGURE 1:**
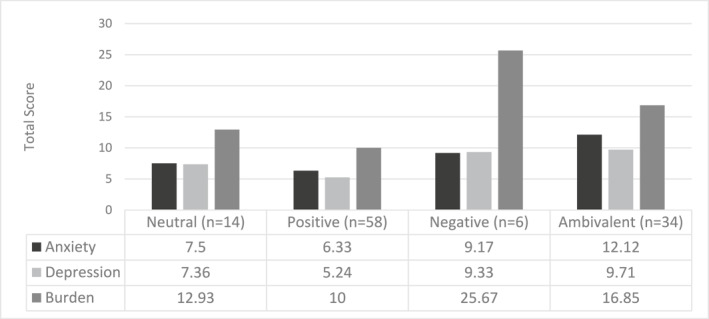
Total anxity, burden, depression scroes by relationships category.

Statistically significant differences between relationship categories were seen in anxiety (*F* = 4.087, *p* < 0.001, *η* ^2^ = 0.183), depressive symptoms (*F* = 4.872, *p* = 0.003, *η* ^2^ = 0.119), and burden (8.829, *p* < 0.001, *η* ^2^ = 0.197). Figure 1 shows outcome data plotted by group. Post‐hoc analysis shows that anxiety is statistically significantly higher in caregivers in the ambivalent group compared to neutral (Mean difference = 4.618, SE = 1.746, *p* = 0.046) and positive groups (Mean difference = 5.790, SE = 1.188, *p* < 0.001). Depressive symptoms are statistically significantly higher in caregivers in the ambivalent group compared to the positive group (Mean difference = 4.465, SE = 1.214, *p* = 0.002). Burden is statistically significantly higher in caregivers in the negative group compared to neutral (Mean difference = 12.738, SE = 4.219, *p* = 0.016) and positive groups (Mean difference = 15.667, SE = 3.708, *p* < 0.001), and caregivers in the ambivalent group reported significantly more burden than those in the positive group (Mean difference = 6.853, SE = 1.868, *p* = 0.002).

## 4 Discussion

This study represents one of the first studies to focus on two key social roles within the networks of caregivers of patients with brain tumors. Little previous work has focused these critical relationships in the neuro‐oncology context, and the work that is in this area has mostly focused on the patients' social relationships [18, 26, 27]. Our data suggest caregivers identify both confidants and people who contribute to the caregiver's stress. These specific kinds of relationships have been associated with caregiver distress and burden, which may in turn, impact ability to provide high quality care for patients [5, 6].

The vast majority of caregivers in our sample identified a confidant, which suggests that many caregivers likely had opportunities to share important stressors regarding caregiving, such as feelings of guilt or burden associated with their role. The percent identifying a confidant in our sample is higher than that seen in prior work on older adults [28, 29], and in contrast to data suggesting a higher likelihood of loneliness in caregiving samples [30]. However, it is important to note that there were individuals who did not note a confidant, and this study did not explicitly measure feelings of loneliness. More work is needed to determine if the broader support networks of both those with and without confidants are adequate to meet caregiver support needs. Additionally, about a third of caregivers identified an individual contributing social stress. While this proportion is much lower than those identifying a confidant, there may be a bias to attend more to negative stimuli compared to positive stimuli [31]. This suggests that the stress of these individuals may outweigh the support provided by the confidant. More research should be dedicated to identifying strategies to help support caregivers in setting boundaries or otherwise manage conflict or unhelpful relationships.

While those who are married most often identify a spouse as a confidant [32], our sample's reliance on friends as confidants aligns with prior work focused on confidants related to marital issues [29] because many of our participants were caring for a spouse. While spouses often engage in dyadic coping and rely on each other for support during stressors, dyadic coping can be additionally complicated when coping with an issue like cancer caregiving. It has been well established that caregivers may hold back emotional disclosure about their stress, or engage in protective buffering to avoid burdening the patient [33]. This may be compounded when the stress comes from caregiving, and the person you may normally cope with may be part of the stress [34]. In particular, patients with brain cancer may experience personality and behavior changes and require significant care, which can result in uncomfortable caregiver feelings about changes or perceived loss of the relationship [35]. These difficult changes, in addition to a changing relationship, may explain why in our study nearly half of people identified as stressful were patients who were the caregiver's spouse.

When a spouse is no longer able to provide the same kind of support, other family members or friends may be important to fill the gap. Caregivers may engage in adaptive strategies of identifying new confidants to manage their current stressors. In our study, friends were the most commonly identified confidant relationship by caregivers. While family members are often seen as critical for support due to the obligation that can be associated with that bond, the obligation also means that it can be difficult to eliminate these relationships if they are stressful [19]. The voluntary nature of friendship can mean that those friendship bonds that survive over many years may be more useful, stronger, or have less co‐occurring negativity than familial bonds. There may also be more opportunity to seek out specific experiences in a broader network of friendships that may be useful for peer support [36]; for example, it may be more likely to find a friend versus. a family member with experience caregiving. These relationships can grow to be more important when friends bond over shared experiences [37].

In assessing the association of critical relationship categories and caregiver outcomes, our findings align with theory [3], such that having a confidant and no person providing stress is associated with lower levels of distress and burden. Conversely, in line with prior research [38], we see interactive effects of these types of relationships, such that caregivers who identified both a confidant and a person contributing stress were more likely to experience anxiety, depression, and burden compared to the confidant only group. The presence of a confidant does not seem to mitigate the negative impact of the presence of a person contributing stress for anxiety and depression, and may only somewhat mitigate the impact of a stressful person for burden. Instead, the presence of a confidant may highlight the missteps or lack of support from the person contributing stress. This may be due to negativity bias, where outsized attention is paid to negative constructs in comparison to positive. Additionally, those contributing high stress may be in closer relationships, either in terms of proximity or intimacy, making it more challenging to create boundaries or distance. For example, many of those identified as contributing stress in our study were spouses, and the frequent interactions and emotional intimacy embedded in this relationship may make it difficult to reduce the number or impact of stressful interactions. Other research suggests that in addition to impacts on psychological health, these complex relationships may also have physical health implications [39], which could be explored in future work.

### 4.1 Clinical Implications

Clinicians should be aware of the social context of neuro‐oncology caregivers, particularly between the patient and caregiver. Interestingly, patients, especially spouses, frequently were nominated as either a confidant or a source of stress for caregivers. Clinicians who understand the interpersonal dynamics can encourage the positive aspects of patient‐caregiver confidants, while also offering tools to patients who may be offering particular stress to caregivers. Patient‐related interpersonal stress identified in clinical interactions may be an indication that additional caregiver psychosocial support is needed. Psychologists, social workers, or navigators could help caregivers manage their relationship with the patient and other relationships to capitalize on strengths and reduce stressors.

### 4.2 Limitations

Several limitations to this study exist. A more diverse sample may uncover more individual characteristics of caregivers associated with reporting having different critical relationship roles. Our data are also cross‐sectional. It is possible that caregiver distress and burden shape the interactions caregivers have with their broader networks and impact their perception of network members as confidants or as causing stress. This could only be examined with longitudinal data. Finally, more complex social network analysis could provide more insight to the social context of caregivers of patients with brain tumors, particularly the impact of support or stress from others, including multiple confidants or people contributing stress, as well as the potential interdependent effects of support and strain experienced by the patient.

## 5 Conclusion

Although the majority of caregivers of people with brain tumors have confidants, who can serve an important role in providing critical support, many caregivers also identify people in their social networks who provide stress. The presence of these individuals can negatively impact caregiver well‐being in a way that is not counterbalanced by the co‐presence of a confidant. Clinicians, such as psychologists, social workers, and navigators, should be aware of the unique needs of caregivers and how members of their social networks, particularly those filling critical relationship roles, can impact their perceptions of distress and burden. There is a particular need to help caregivers manage potential stressful relationships that can exacerbate existing stress from caregiving.

## Author Contributions

M.R., M.M.B., B.J.Z., K.J.W. contributed to study conception and design. Data preparation and review were performed by M.R. and D.W. Data analysis was performed by M.R. The first draft of the manuscript was written by M.R. All authors commented on previous versions of the final manuscript. All authors read and approved the final manuscript.

## Funding

This work was supported by the National Cancer Institute, under award R01CA236034. This work was also supported by the Biostatistics and Bioinformatics Shared Resource at the H. Lee Moffitt Cancer Center and Research Institute, a National Cancer Center Institute designated Comprehensive Cancer Center (P30CA76292, PI: Cleveland).

## Conflicts of Interest

The authors declare no conflicts of interest.

## Data Availability

De‐identified data from this study are not available in a public archive. De‐identified data from this study will be made available (as allowable according to institutional IRB standards) by emailing the corresponding author. Analytic code used to conduct the analyses presented in this study are not available in a public archive. They may be available by emailing the corresponding author. Materials used to conduct the study are not publicly available.
